# Sedentary behaviour and activity patterns of older adults in an acute hospital setting: An exploratory study

**DOI:** 10.1177/03080226251375309

**Published:** 2025-10-18

**Authors:** Laura J Brown, Samantha J Warne, Chun Ngai Liu, Jemma Hutchins, Joanne McVeigh, Craig Thompson, Kristie J Harper

**Affiliations:** 1 Curtin School of Allied Health, Occupational Therapy, Curtin University, Perth, AU-WA, Australia; 2 Sir Charles Gairdner Osborne Park Health Care Group, Perth, AU-WA, Australia; 3 EnAble Institute, Curtin University, Perth, AU-WA, Australia

**Keywords:** accelerometry, aged, functional status, hospitals, occupational therapy

## Abstract

**Introduction:**

Hospitalisation is a period of low physical activity, particularly for older adults. Objective measurement of activity levels is essential to evaluate the impact of interventions to maintain or improve function.

**Methods:**

A prospective exploratory study examining patterns of sedentary behaviour and physical activity using an accelerometer-based activity monitor (activPAL™) in an acute hospital setting. A secondary objective was to review patient and staff acceptability of accelerometer use and views on the barriers and opportunities to maintaining function.

**Results:**

Forty-nine adults were recruited with a mean (SD) age of 83.0 (9.64) years (67% females). The mean (SD) time spent sitting or lying was 19.9 (5.03) hours with patients taking 657 (820) steps per day. There were no significant differences between steps per day for different genders (*p* = 0.78), age groups (*p* = 0.77) or diagnostic groups (*p* = 0.60). There was a moderate correlation between the daily number of steps and the Functional Independence Measure (*r* = 0.37, *p* < .05). Eighty-six per cent of patient’s found accelerometer use acceptable compared to 50% of staff.

**Conclusion:**

This study has provided rich characterisation of activity patterns in hospital, where low levels of activity were identified. These findings can support future occupational therapy functional maintenance initiatives.

## Introduction

Approximately 60% of older adults in hospital develop new dependence in activities of daily living (ADL) during a hospital stay, and only 68% recover to their previous level of function 6 months after discharge (Li et al., 2020). Older people in hospital are at risk of developing iatrogenic conditions independent of the primary reason for admission resulting in complications such as delirium, falls and hospital acquired functional decline (Jasper et al., 2020). One main reason for functional decline is excessive periods of prolonged uninterrupted bouts of sedentary behaviour or reduced activity, (Jasper et al., 2020) which has been shown to result in increased loss of muscle mass, (Kortebein et al., 2007) increased risk of death, (Brown et al., 2004) increased length of hospital stay, (McCullagh et al., 2016) and functional decline (Hartley et al., 2020).

Positive health outcomes in older adults are directly linked to engagement in activity during and after hospitalisation (Floegel et al., 2019; Padula et al., 2009). Studies have reported that activity levels of older adults in hospital are very low (Li et al., 2020). Previous studies have found participants spent an average of 99% of their day either lying or sitting and a little more than 1% of the day either standing or walking (16 minutes per day; Davenport et al., 2015; Grant et al., 2011). In comparison, a recent systematic review (Blackwood et al., 2022) exploring older adult movement in the community reported daily steps ranging from 864 to 15847 steps per day, with sedentary time at 62.5%, highlighting the reduction in activity in hospital settings resulting in poor health outcomes.

Measurement of activity levels of older inpatients is essential to evaluate the impact of interventions to maintain or improve activity levels and to determine associations between activity in hospital and other health-related outcome measures (Lim et al., 2018). Measures of activity in hospital have traditionally been completed via observation, behavioural mapping, use of tools such as the Physical Activity Scale for the Elderly (PASE; Washburn et al., 1993) or through use of pedometers. Recently, objective measurements using body-worn single sensor activity monitors have been applied, enabling continuous monitoring of activity over time (Davis and Fox, 2007; Norvang et al., 2018). A systematic review in 2018 identified acceptable ways in which to measure activity in older adults in hospital (Lim et al., 2018). Eighteen studies were identified, and the activPAL™ was highlighted as useful (Lim et al., 2018). The activPAL™ is a thigh-worn device that uses accelerometer-derived information about thigh position to determine the time patients spend sitting/lying, standing, stepping as well as their step speed, step counts and postural transitions (PAL Technologies Ltd, 2023).

This study aimed to use the activPAL™ to examine patterns of sedentary behaviour and activity in older adults in hospital. The first objective was to describe sedentary behaviour (measured by sustained body posture) and activity (measured by movement intensity) in an acute hospital setting. The second objective was to engage with stakeholders and review patient and staff acceptability regarding use of the activPAL™ and views on the barriers and opportunities for increased activity in hospital. The third objective was to examine the relationships between sedentary behaviour and activity with an existing functional measure (Functional Independence Measure [FIM]) (Dodds et al., 1993). Functional decline in hospital is an enduring issue and objective measurement is not routine practice in occupational therapy. Being aware of the extent of sedentary behaviour and effective measurement are essential first steps to planning effective occupational therapy functional maintenance initiatives aimed at reducing sedentary behaviour (Jasper et al., 2020). Additionally, understanding our staff and patient views regarding objective measurement, device use and activity in hospital is crucial to support development of translatable interventions.

## Method

### Study design and setting

This prospective exploratory study was completed at an adult tertiary hospital. All participants provided written informed consent. All data were de-identified and analysed in aggregate form. Ethical approval was obtained from Sir Charles Gairdner Osborne Park Health Care Group Human Research Ethics Committee (HREC) (RGS0000005086) with reciprocal approval from Curtin Univeristy HREC (HRE2022-0075) in 2022. Reporting adheres to the STROBE statement for observational studies (Von Elm et al., 2007).

### Study population

This study included two key groups consisting of hospital inpatients and clinical staff. The study was conducted across three acute general medical hospital wards over a 4-month period (February 2022–June 2022). The inclusion criteria for patients consisted of older adult inpatients (aged ⩾55 years), who had mild to no cognitive impairment (⩾6 on Abbreviated Mental Test [AMT-10]) (Jitapunkul et al., 1991) and had a predicted inpatient length of stay (LOS) of at least 2 days on the general medical ward. An AMT-10 score ⩾6 was selected, in consultation with the ward Geriatrician, to support recruitment of participants with normal cognition and those with mild cognitive impairment who could wear an activPAL™ device. Given the acute hospital setting, a minimum length of stay of two days was selected to allow sufficient time for exploratory monitoring of physical activity using the activPAL™, ensuring meaningful data capture within the constraints of short inpatient admissions. Patients who were unable to provide informed consent or unable to speak English (if no professional interpreter was available) were excluded from the study.

Consecutive eligible patients were identified at ward admission by either the senior occupational therapist or physiotherapist on the three participating general medical wards. The research assistant was informed, who approached the patient regarding participation. Following provision of informed consent, patients were fitted with an activPAL™ accelerometer by the research assistant.

Hospital staff were eligible to participate in the written feedback survey if they met the inclusion criteria of being an occupational therapist, physiotherapist, nurse or allied health assistant, working with patients on the included wards. They were again recruited by a research assistant. As this was an observational study, a formal sample size calculation was not appropriate as definitive effectiveness was not being assessed (Lancaster et al., 2004). A pragmatic sample size of 50 patients and 10 staff were planned in line with the recruitment period available, research funding and number of activPALs™ available.

### Variables, data sources and measurement

#### Accelerometer

The activPAL™ is a valid measure of standing, sitting and lying and has displayed reasonable accuracy in older adults with a range of walking (Bourke et al., 2019; Hergenroeder et al., 2018). The research assistant applied the activPAL™ to the patients’ less affected or dominant leg. The activPAL™ was attached to the midline of the anterior upper-thigh, one-third of the way between the thigh and the knee. The patient was seated during the attachment. Skin was prepared with a Cavilon wipe and air-dried. DuoDerm was placed on the thigh followed by the waterproof activPAL™ and attached with Fixomoll, which did not touch the patient’s skin as per local pressure and skin management guidelines.

The activPAL™ was covered by an additional waterproof dressing (Tegaderm™). The activPAL™ could be removed due to special circumstances which included radiography, magnetic resonance imaging (MRI) scans or have a dressing changed. If any of these events occurred, the research assistant documented the reason for removal and reattached the activPAL™. The device was removed at least 2 days after application and prior to hospital discharge. Valid data was considered two plus days, whereas participants with <2 days of data were considered to have insufficient activity data. The data were downloaded from the activPAL™, cleaned and de-identified.

#### Social and clinical descriptors

Patient demographic, social and baseline clinical descriptors were recorded at recruitment by the research assistant on the ward with the participant. General demographics consisted of gender, age, living situation (i.e. with partner, with family, or alone) and service use. Service use was defined as formal care (i.e. paid care providers) or non-formal care (i.e. family or unpaid carers) to assist with domestic or personal activities of daily living.

The Charlson Comorbidity Index (CCI;Charlson et al., 1987) was used to describe burden of chronic disease. The CCI is a weighted index where a higher score indicates a greater burden of comorbidities and is associated with increased mortality risk (Charlson et al., 1987). The Clinical Frailty Scale (CFS;Rockwood and Theou, 2020) was used to evaluate a patient’s level of frailty using a nine-level scale ranging from very fit to terminally ill. The FIM was used to measure the patient’s level of function at recruitment (Dodds et al., 1993). The FIM score was calculated from 18 items using a seven-point scale to determine the patient’s level of functional independence (Dodds et al., 1993). A higher score (total of 126) indicated better function (Dodds et al., 1993). Two staff completed the FIM and were accredited in tool use.

A patients’ mobility was defined as either independent, independent with aid or required assistance. Number of falls in the past 12 months was recorded. Additionally, number of hospital acquired complications (HAC) including delirium, pressure injuries (PI) and falls during the admission and hospital LOS were noted upon patient discharge.

#### Patient survey

All patients were asked to participate in a short-written survey to establish acceptability of the activPAL™ and barriers and opportunities for activity in hospital. This consisted of seven open-ended questions (See Appendix). This survey was completed upon removal of the activPAL™ with the research assistant.

#### Staff survey

The staff survey explored their perceptions regarding activPAL™ use and the barriers and opportunities to support patient activity in the hospital environment. This consisted of one yes/no question, identified the clinician’s profession and included five open-ended questions (See Appendix). Both the patient and staff survey were piloted prior to use with a group of two staff and two consumers to support usability and data collection.

#### Bias

All data were collected by an independent research assistant. Patient activity patterns may also have been impacted by the Hawthorne effect in which the patient follow-up or attention may have impacted activity behaviour. To mitigate this, daily activPAL™ site checks were kept to a minimum and were built into standard care.

### Data management and processing

Demographics including age, social and baseline clinical descriptors and survey responses were entered into Research Electronic Data Capture (REDCap; Harris et al., 2009). The activPAL™ software was used to download and export the activPAL™ data for analysis. ActivPAL’s CREA algorithm (v1.3) was used to process data; the algorithm was used to provide wear time, valid wear time days, non-upright/ sedentary time (sitting and lying time), primary lying time (to estimate sleep time), posture changes (sit- stand transitions) and steps/day (PAL Technologies Ltd, 2023). The processed data were also checked visually (by creating heatmaps of the data) (Winkler et al., 2016), to ensure correct classification of sleep and waking behaviour.

### Statistical analysis

Descriptive and univariate statistics were used to analyse the data in relation to social and clinical descriptors. To determine physical activity levels, the patients’ volumes and patterns of activity behaviours measured by the activPAL™ were extracted including daily steps taken, time spent in a seated and lying position, as well as the number of postural transitions made (i.e., sit to stand or vice versa) per day. Normality of the data were assessed using the Komologrov–Smirnov test. Parametric testing using Pearson’s *r* was conducted, and for normally distributed data, the mean and standard deviation were reported in conjunction with the correlation statistic. Where data were non-normally distributed, non-parametric testing using Spearman’s *Rho* was conducted and the median and interquartile range (IQR) were reported. Correlations between the FIM score, age, CFS, mean number of daily steps, and mean duration of changes in postures were completed. Activity and sedentary behaviour differences between genders, age groups and presenting diagnostic groups were explored using *t*-tests, Mann–Whitney *U* Test or Kruskal–Wallis ANOVA, dependent on meeting data analysis assumptions. The Statistical Package for Social Sciences (SPSS) Version 28.0 was used to conduct the statistical analysis and *p* values ⩽0.05 were considered statistically significant.

The survey findings were reviewed to explore the acceptability of objective measurement, barriers and opportunities for activity. Raw patient responses to open-ended questions were reviewed using summative content analysis, whereby key words were identified and quantified (Hseih and Shannon, 2005; McKenna et al., 2017). Three researchers supported this process, reviewing the survey feedback independently. The researchers reviewed the text to identify the semantic meaning units that were then converted to codes. The researchers reviewed their independent codes, and any discrepancies were discussed until a consensus was reached. Saturation was defined as the point at which no new codes were emerging in the feedback. The codes were categorised and quantified to establish frequency counts.

## Results

In this exploratory study, 61 patients were approached to participate. Two males and one female declined to participate, and five were excluded due to cognitive impairment. Fifty-three patients consented to participate; however, four patients were excluded from the study due to insufficient wear time and data, that is, less than two days. Overall, 49 patients were included in the final data analysis. Sixty-seven per cent were female, aged between 73 and 93 years old (Table 1). The median hospital LOS was 12 days (IQR = 17). Approximately half of the patients lived alone (51%, *n* = 25). Most patients could walk, with 73% (*n* = 36) being independent with aid and 4% (*n* = 2) requiring assistance to mobilise. Most patients used services at home to assist with domestic or personal activities of daily living, with 76% (*n* = 37) being formal services (paid care providers), 10% (*n* = 5) being non-formal (family or unpaid carers) and 14% (*n* = 7) of patients used no services. The main reason for hospitalisation was a fall (35%, *n* = 17) or other diagnoses (33%, *n* = 16) including urinary tract infection (UTI), headaches and cancer-related complications. Additionally, 22% (*n* = 11) of patients experienced a HAC including delirium (6%, *n* = 3), PI (10%, *n* = 5) or a fall during their hospital admission (6%, *n* = 3).

**Table 1.** Demographic characteristics of the patient participants and activPAL™ output (*n* = s49).

Variables	Total *n* (%)
Females	33 (67)
Males	16 (33)
Age (years), mean (SD)	83 (10)
Living situation	
Alone	25 (51)
With partner	13 (27)
With family	11 (22)
Service use	
None	7 (14)
Informal	5 (10)
Formal	37 (76)
Presenting diagnosis	
Fall	17 (35)
Pain	10 (20)
Respiratory system	3 (6)
Digestive system	3 (6)
Other (Urinary Tract Infection, Cancer, etc.)	16 (33)
Comorbidities	
Hypertension	25 (51)
Respiratory system	11 (22)
Arthritis	9 (18)
Ischemic heart disease	7 (14)
Charlson Comorbidity Index, median (IQR)	5 (2)
Abbreviated Mental Test – 10, median (IQR)	10 (2)
Clinical Frailty Scale, median (IQR)	5 (1)
Hospital length of stay (days), median (IQR)	12 (17)
Fallen in past 12 months	32 (65)
Mobility level, *n* (%)	
Independent	11 (22)
Independent with aid	36 (73)
Assistance required	2 (4)
ActivPAL^™^ Data	
Total number of days worn (sum of all participants)	106
Days worn, mean (SD)	2 (1)
Total wear time (minutes/day), mean (SD)	1300 (262)
Daily wear time (minutes/day), mean (SD)	877 (262)
Daily steps (steps/day), mean (SD) [min-max]	657 (820) [5–4565]
Daily positions/posture change, mean (SD)	
Lying position (estimated sleep time), (minutes/day)	423 (319)
Non-up right position (sitting and lying time during day), (minutes/day)	772 (279)
Posture change (sit – stand), (numbers/day)	13 (7)

## Activity and sedentary behaviour patterns

Collectively, the patients wore an activPAL™ for a total number of 106 days, with a mean wear time of two (SD = 1) days per patient (Table 1). The device was fitted on a median of day five (IQR = 14) during a patient’s hospital LOS (total hospital LOS was a median of 12 days (IQR = 7)) when they were admitted to the general medical ward. Patients spent a mean of 10 (SD = 10) mins/day stepping and accumulated a mean of 657 (SD = 820) steps/day. Patients had a mean of 13 (SD = 7) posture changes (sit to stands transitions/day). Patients spent a mean duration of 423 (SD = 319) mins/day lying down in bed (estimated sleep time) or in a non-up right position (seated/lying in bed awake) for 772 (SD = 279) mins/day. Overall patients were sedentary (primary lying time + non-up right position) for approximately 20 hours or 83% of their day. There were no significant differences in sedentary behaviour as per time lying between genders (*p* = 0.979), age groups (*p* = 0.758) or presenting diagnoses (*p* = 0.569) (Table 2). Additionally, there were no significant difference in non-upright time again between genders (*p* = 0.549), age groups (*p* = 0.230) or presenting diagnoses (*p* = 0.556).

**Table 2.** Volumes and patterns of activity behaviours as measured by the activPAL™ stratified by sample characteristics (*n* = 49).

Sample Characteristics	Daily steps (median steps/day, IQR)	*p* Value
Gender		
• Male (*n* = 16)	490 (571.5)	0.777
• Female (*n* = 33)	427 (610.7)	
Age		
• 70–80 years (*n* = 14)	540 (1166.7)	0.774
• 81–90 years (*n* = 19)	325 (581.0)	
• 90+ years (*n* = 13)	446 (496.5)	
Presenting diagnosis		
• Fall (*n* = 17)	495 (603.0)	0.599
• Pain (*n* = 10)	409 (943.0)	
• Respiratory system (*n* = 3)	479 (433.5)	
• Neurological (*n* = 6)	488 (2855.3)	

## Relationship between activity and function

**Figure 1.**
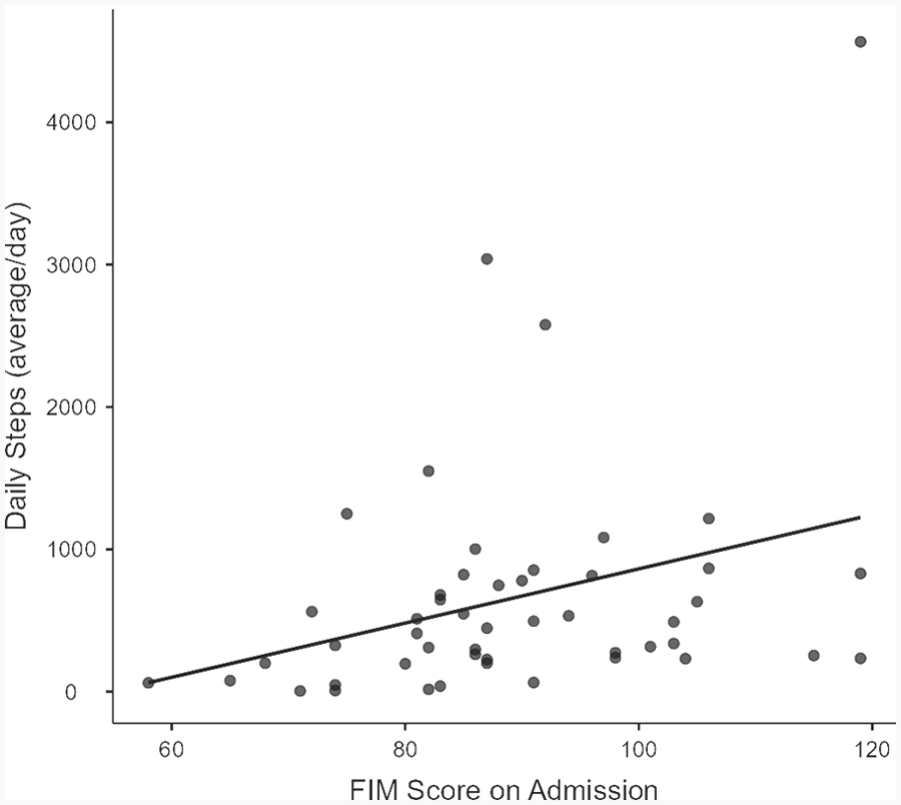
Correlation between Functional Independence Measure (FIM) score and mean daily steps (*r* = 0.37, *p* < 0.05).

**Figure 2.**
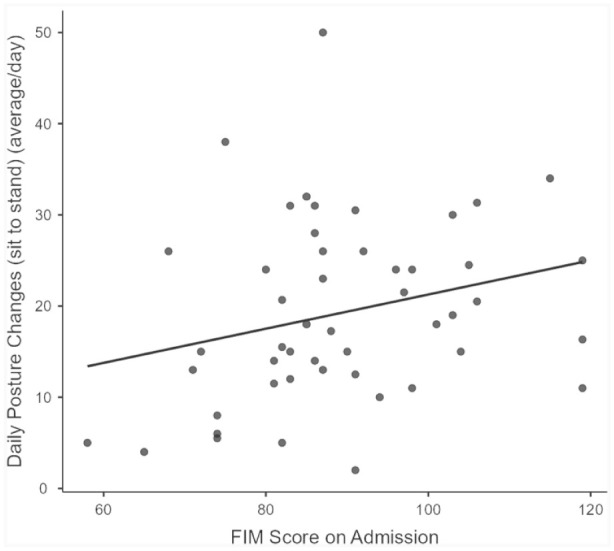
Correlation between Functional Independence Measure (FIM) score and mean daily posture changes (sit to stand; *r* = 0.30, *p* < 0.05).

Age was not significantly correlated with the FIM score (*r* = −0.19, *p* = 0.22), and the correlation between frailty (CFS score) and the FIM score was *r* = −0.54, *p* < 0.05. Additionally, the correlation between the daily number of steps and the FIM score was *r* = 0.37, *p* < 0.05 (Figure 1). Furthermore, the correlation between the mean number of patient posture changes (sit to stand) and the FIM score was *r* = 0.30, *p* < 0.05, (Figure 2). Neither time spent lying down (*r* = 0.28, *p* = 0.05), nor time spent in the non-up right position (*r* = -0.22, *p* = 0.13) was associated with the FIM score.

## Acceptability of the ActivPAL™

Out of the 49 patients, 44 (90%) agreed to complete the survey with saturation in codes identified after patient 30. Eighty-six per cent (*n* = 38) of patients reported that the device was comfortable to wear. Four patients experienced slight discomfort with the device being removed, with hair being pulled from the sticky adhesive. Ninety-one per cent of patients (*n* = 40) agreed that it was important for them to remain active while hospitalised. Seventy-seven per cent (*n* = 34) of patients did not believe that wearing activPAL™ made them more active, six patients stated, “*they forgot it was there*,” while 11 patients reported they either needed assistance to move or were in too much pain to move.

Eleven hospital staff completed the survey: four occupational therapists, three physiotherapists, two nurses and two allied health assistants with a 68% response rate supporting saturation of feedback after survey eight. All staff agreed that the activPAL™ was accepted by patients. However, six of the hospital staff believed it was not feasible to use the activPAL™ in the hospital setting because two were lost (coming off in the bed or shower). One staff member commented it was not feasible to use the activPAL™ due to the cost; *cost of the activPAL™ is a big consideration and importance of not losing them.* They went on to state *there is the potential for issues with applying and securing the activPAL™*. Six hospital staff believed the activPAL™ made the patients more active, one staff member reporting *in some cases for example intrinsically motivated patients or more mobile patients*. Additionally, the need for real-time data output was highlighted.

### Barriers and Opportunities for Activity in Hospital

Ninety per cent of patients (*n* = 40) were supportive of programs to maintain activity in hospital. Patients identified intrinsic barriers to activity as physical (pain, fatigue and weakness) or psychological (fear of falling). However, most patients identified a greater number of extrinsic barriers to activity. This included lack of purposeful activity, lack of staffing to assist more regularly, falls prevention and management strategies (where patients were concerned with falling or not encouraged to mobilise independently due to falls risk) and feeling *in the way.* They were also aware of the environmental limitations such as decreased bed space, no walking paths, no activity rooms and unable to access outdoors (Table 3).

**Table 3.** Survey results consisting of patient (*n* = 44) and staff (*n* = 11) identified barriers and opportunities for activity in the hospital setting.

Participant	Barriers	Opportunities
Patients	Requiring assistance, limited mobility, pain (*n* = 26, 59%)	Better access to activity (*n* = 25, 57%)
	Decreased staffing, availability (*n* = 30, 68%)	More equipment, ‘chair stops’ (*n*=20, 45%)
	‘Nothing to do’, boredom (*n* = 22, 50%)	Outdoor access (*n* = 18, 41%)
	Environmental limitations, decreased space, ‘in the way’ (*n* = 34, 77%)	Reminders, individually tailored, and social aspect is valued (*n* = 21, 48%)
Staff	Requires resources (*n* = 8, 73%)	Team approach, better use of therapy assistant support (*n* = 7, 64%)
	Decreased staffing, availability (*n* = 9, 82%)	Use of groups (*n* = 5, 45%)
	‘Too risk adverse’, lack of patient empowerment (*n* = 6, 54%)	Creating space, providing a ‘reason to leave the bedspace’ (*n* = 6, 54%)
	Environmental limitations, decreased space (*n* = 9, 82%)	Better use of family or caregivers (*n* = 5, 45%)

Opportunities identified by patients to support activity included the creation of activity opportunities, that is, walk to get the paper, use kitchenette, provision of gym equipment and better ward set up such as increasing the number of chair stops available on the ward to increase confidence during walking. Patients also reported needing reminders to support their activity participation, would like tailored recommendations and again highlighted the importance of social engagement as a motivating factor.

One hundred per cent of hospital staff (*n* = 11) agreed it was important to keep older adults in hospital active. Staff identified barriers to activity in hospital were similar to patient findings such as awareness of decreased space, environmental limitations and decreased staffing. It was also highlighted that staff may be too risk-adverse, that is, restricting patient activity to avoid possible falls and that additional resources are required to support activity participation. Possible opportunities identified by staff included strong multidisciplinary team support, opportunity to increase allied health assistant support, use of inpatient groups, need to review the ward space and create a reason for patients to leave the bedspace and possible better use of family (Table 3).

## Discussion and implications

This study achieved its primary aim of measuring activity in an acute hospital setting and engaging with stakeholders regarding objective activity measurement and the barriers and opportunities for activity in hospital. The current study identified low levels of activity in this population, with patients spending an average of 10 minutes per day stepping. The activity levels were higher than previously observed whereby older adults on an acute geriatric ward were found to spend on average seven minutes per day walking (Villumsen et al., 2015). This disparity may be a result of the attachment time of the monitor in the different studies, with the previous study attaching the monitor on hospital admission versus in our study the monitor was attached at ward admission, closer to discharge when patients may have been more mobile. Our study confirmed previous research findings in which low levels of activity in hospital were noted (Jasper et al., 2020; Rojer et al., 2023; Villumsen et al., 2015).

The current study revealed higher levels of frailty were associated with poorer functional independence in older adults in acute care. Additionally, patients with better function took more steps compared with those with poorer levels of function. While a correlation between activity and function was expected, these results highlight the value of utilising the FIM and CFS in identifying patients in an acute care setting at risk of functional decline in the absence of routine use of objective measurement. This echoes the findings of prior research, which show correlations between increased patient frailty, reduced FIM scores and associated reductions in activity (Kawryshanker et al., 2014).

This study explored patient’s acceptance regarding use of an activPAL™ in the acute hospital setting. Eighty-six per cent of patients reported the device was comfortable to wear. Common themes regarding comfort are echoed in other studies (Berendsen et al., 2014; Grant et al., 2008; Radtke et al., 2021). Patient amenability to wearing the activPAL™ combined with staff reports that all patients accepted the device indicate high levels of acceptability. These findings are significant as they demonstrate acceptance of using accelerometers to track activity in hospital and could support objective measurement of clinical interventions aiming to reduce hospital functional decline.

This study has ascertained stakeholder views regarding the barriers and opportunities to support activity in hospital which is crucial to the development of interventions targeting activity and functional decline in this population (Jasper et al., 2020). The barriers identified by staff were similar to previous research and included environmental and institutional factors (care processes, prescribed immobility, hospital culture, limited time and staffing; (Geelen et al., 2021). Ninety per cent of patients indicated that they were supportive of activity programs to maintain their independence, similar to other research, which indicated that hospitalised older adults consider functional mobility as essential in maintaining quality of life and their sense of well-being (King et al., 2021).

Many patients in this study felt passive regarding their activity while in an acute care hospital setting reflected as feeling *in the way* and needing staff assistance. This sentiment is echoed by previous literature where some patients felt completely dependent on staff for engagement in activity (King et al., 2021). They identified strategies such as the provision of education and support, for example, how much and what type of activity they should engage in, use of visual reminders, to facilitate independence (King et al., 2021).

This study provides valuable evidence that although the intrinsic motivation of hospitalised older adults to engage in activity is plentiful with 91% indicating activity was valued, it is often quelled by extrinsic physical, environmental and institutional factors with more extrinsic barriers identified compared to intrinsic barriers (Table 3). It was also not reflected in the accelerometer output with low activity levels being achieved. Furthermore, while barriers to activity in this setting are easily identified, their reoccurrence in the literature suggests no initiatives to address them have yet been designed or implemented (Jasper et al., 2020). This study provides stakeholder input that could support the development of future occupational therapy initiatives to address functional decline in hospital.

### Limitations

This study was completed at a single site. Future studies would benefit from expanding the sample size and including patients with cognitive impairment to further increase the degree to which study findings could be generalised to broader populations. Additionally, data on ethnicity could be collected in future research. Due to the rapid nature of our acute care setting (ward average LOS of 4.6 days), the mean device wear time was two days. Subsequent research in the acute setting should consider fitting patients with devices earlier in their admission to increase device wear time. More females participated in this study (67%) impacting on the generalisability of the findings. Consideration of gender differences and preferences would be essential to design interventions to support activity participation in hospital. Recruitment occurred across different wards, and this was not controlled for during statistical analysis, which could be considered in future research.

### Clinical implications

This study may be used to inform future research to address activity and sedentary behaviour in a hospital setting, and it has applicability to activity measurement in occupational therapy, which is limited in current research. It demonstrates how accelerometers can be incorporated into an acute care setting as an outcome measure. Accelerometer use by occupational therapists may offer objective activity measurement that extends beyond tracking movement, also enabling insights into time spent in specific postures such as sitting or lying, which can support pressure care management for example.

Additionally, it has captured patient and staff views on activity in hospital that can be used to develop future functional maintenance initiatives. As both staff and older adults identified primary barriers to activity as institutional and environmental, occupational therapists have a role in the development of initiatives to resolve them (i.e. modifying hospital environment, funding allocation and staffing). Understanding change from patient baseline sedentary levels could also be reviewed. This project was supported by research staff who assisted with device management, further research would be required to explore integration of the accelerometers into standard clinical practice.

## Conclusion

This study has provided rich characterisation of activity patterns in hospital where low levels of activity were identified. Additionally, stakeholder views regarding activity measurement, barriers and opportunities for activity in hospital have been identified. These findings could be used to support future occupational therapy functional maintenance initiatives and inform therapeutic activity targets for adults in hospital.

**Key findings**

- Older adults in hospital were sedentary for 83% of the day.
- Accelerometer use was well accepted by patients and staff, despite feasibility concerns.
- The FIM significantly correlated with activity levels.

**What this study adds**

Low levels of activity were found in older adults in hospital. Accelerometers can be used by occupational therapists to support objective activity measurement in hospital.

## Appendix

### Patient survey questions

1) How do you feel about wearing an activPAL™ for activity monitoring in hospital?
2) Do you think wearing the activPAL™ made you more conscious of your own activity levels?
3) Did wearing the activPAL™ make you want to move more?
4) Is it important for you to remain active in hospital?
5) Could we support adults in hospital to remain active? If yes, how?
6) What are the barriers to activity in hospital?
7) What are the enablers to activity in hospital?

### Staff Survey Questions

1) In your opinion, were the use of activPAL™ accepted by patients?
2) Were there any issues regarding use of the activPAL™ on the ward?
3) In your opinion, do you think use of activPALs™ impacted on patient activity levels?
4) Do you feel it is important for adults to remain active in hospital?
5) What are the barriers and enablers to support older adult activity in hospital?
